# Node Calibration in UWB-Based RTLSs Using Multiple Simultaneous Ranging

**DOI:** 10.3390/s22030864

**Published:** 2022-01-23

**Authors:** Shashi Shah, La-or Kovavisaruch, Kamol Kaemarungsi, Tanee Demeechai

**Affiliations:** National Electronics and Computer Technology Center, National Science and Technology Development Agency, Phahonyothin Road, Pathumthani 12120, Thailand; la-or.kovavisaruch@nectec.or.th (L.-o.K.); kamol.kaemarungsi@nectec.or.th (K.K.); tanee.demeechai@nectec.or.th (T.D.)

**Keywords:** antenna delay, localization, node calibration, two-way ranging, ultra-wideband

## Abstract

Ultra-wideband (UWB) networks are gaining wide acceptance in short- to medium-range wireless sensing and positioning applications in indoor environments due to their capability of providing high-ranging accuracy. However, the performance is highly related to the accuracy of measured position and antenna delay of anchor nodes, which form a reference positioning system of fixed infrastructure nodes. Usually, the position and antenna delay of the anchor nodes are measured separately as a standard initial procedure. Such separate measurement procedures require relatively more time and manual interventions. This paper presents a system that simultaneously measures the position and antenna delay of the anchor nodes. It provides comprehensive mathematical modeling, design, and implementation of the proposed system. An experimental evaluation in a line-of-sight (LOS) environment shows the effectiveness of the anchor nodes, whose position and antenna delay values are measured by the proposed system, in localizing a mobile node.

## 1. Introduction

The use-case applications of real-time localization systems (RTLSs) are essential across various fields, such as in healthcare, industries, surveillance, crisis management, and so on [1,2,3]. Global positioning system (GPS)-based RTLSs are used in a wide range of applications; for example, they are commonly used for automobile navigation, as they provide global coverage and an average accuracy of 1–5 m when using consumer-grade devices [4,5]. However, their applicability is limited to outdoor RTLSs, since they require LOS with GPS satellites [1,5]. The advent of wireless technology and the large-scale proliferation of wireless communication devices have opened doors for several indoor RTLS applications in recent years [6,7]. For such indoor RTLS applications, the global navigation satellite system is, in general, not suitable due to the stringent requirement of high localization accuracy [8].

An indoor RTLS facilitates localization of mobile assets in an indoor environment [9], where a reference positioning system of fixed infrastructure nodes, known as anchor nodes, is needed, and it is to be used to localize other mobile nodes within its coverage area. Usually, calibration of these anchor nodes is performed as a standard initial procedure to determine their positions [10]. This can be obtained by external measurement equipment (such as a laser or surveyor) or by the system itself, i.e., auto-calibration. The former procedure can be expensive and is often time-consuming. However, auto-calibration can automate and simplify the calibration procedure with no requirement of additional measurement equipment, thus making it far less tedious.

Most indoor RTLSs are based on radio communication technologies, such as wireless local area networks (WLANs), Bluetooth, radio-frequency identification, infrared, and UWB [7,11]. WLANs and Bluetooth do not require any dedicated infrastructure, while the other three technologies require dedicated infrastructure for their operations. This characteristic of WLANs and Bluetooth makes them common and popular candidates for indoor localization because of their use of off-the-shelf mobile devices and relatively low additional infrastructural cost [12,13,14]. However, they are less accurate and insufficient for applications with high accuracy requirements. As an example, a WLAN-based localization method presented by Yang et al. [15] achieves an accuracy of roughly 70 cm. As shown by the comparisons carried out on indoor positioning technologies in [11,16], UWB is particularly interesting due to its capability of providing high accuracy, material penetrability, coverage, and scalability. These capabilities were introduced and supported in IEEE 802.15.4a as UWB physical layer (PHY) [17,18] and were later integrated into the IEEE 802.15.4 standard as UWB PHY [19]. Recently, in 2020, the IEEE 802.15.4z-2020 standard [20] was introduced, which aims to introduce new capabilities and enhance the already-existing standards for UWB technology. Some of the notable features of the standard are improved ranging, reduction to on-air transmissions, the introduction of simultaneous ranging, and improved timestamp robustness and security.

In this regard, UWB networks are widely used in indoor RTLSs, since they have the advantage of providing accurate timestamp information of the transmitting and receiving signals by the UWB nodes in scenarios satisfying LOS conditions, while complex techniques are needed for non-line-of-sight (NLOS) conditions to obtain accurate results [6]. The timestamp information is, in turn, used to compute the time of flight (TOF) between two UWB nodes, which is usually estimated with two-way ranging (TWR) methods [19,21,22,23,24]. These TWR methods usually focus on minimizing the TOF error between the UWB nodes due to the clock offsets that exist due to imperfections in clock oscillators in physical environments [25,26]. Although the technology estimates the distance between two nodes with centimeter-level accuracy, the correctness of the position of the anchor nodes is crucial for a highly effective UWB-based RTLS. Moreover, another significant aspect that affects the accuracy of the UWB-based RTLSs is the antenna delay of the UWB nodes [27]. It is device-specific and is mainly caused by the underlying analog circuitry that produces a quasistatic bias in the reported timestamp values [27]. The timestamp values reported by the UWB nodes are usually different from the correct timestamp values if the antenna delay values of the UWB nodes are not considered. This leads to an error in the computed TOF and compromises the accuracy of the calculated range unless the reported timestamp values are corrected by taking into account the antenna delay values of the UWB nodes. Hence, to obtain a more accurate UWB-based RTLS, it is necessary to effectively determine the position and antenna delay of the anchor nodes during the calibration process.

The auto-calibration methods present in the literature [28,29,30] require a reference system of predefined coordinates for placement coordination among a fixed number of anchor nodes, which is either done by manually setting their positions or by constraining their placement. Then, other anchor nodes are placed arbitrarily at their specific locations. Hence, these methods rely on strict assumptions on the placement of a set of anchor nodes in the reference positioning system in a predefined order to determine the relative positions of all other anchor nodes within the coverage area. Moreover, although essential, none of these methods comply with the measurement of the antenna delays of the anchor nodes. Several methods specifically dedicated to the measurement of antenna delays of UWB nodes are available in the literature [27,31,32,33]. These methods usually differ in the adopted TWR principles [19,21,23] and the number of TWR sessions performed between a pair of UWB nodes. The overall calibration process of the anchor nodes is relatively long, since the measurements of their position and antenna delay are performed separately in the existing literature. Hence, to exploit the plug-and-play features of an ideal UWB-based RTLS, the calibration process should comply with collectively measuring the position and antenna delay of the anchor nodes as a standard initial procedure. This would simplify the calibration process by making it less time-consuming and requiring fewer manual interventions.

In this paper, we present a novel node calibration system that simultaneously measures the position and antenna delay of anchor nodes in a UWB-based RTLS. During the measurement process, the UWB network is set to comprise anchor nodes located at fixed unknown locations whose position and antenna delay values are to be measured, as well as a mobile node that is placed at predetermined known positions. Then, the position and antenna delay of the anchor nodes are estimated based on the TWR sessions performed between each pair of mobile and anchor nodes. Finally, we evaluate the effectiveness of the proposed system with an experiment that considers RTLS applications such as tracking assets in an indoor office. The results show that the post-calibrated anchor nodes whose position and antenna delay values are measured by the proposed system make more accurate localization of a mobile node. The key attributes of the proposed system are as follows.

- It simultaneously measures the position and antenna delay values of anchor nodes in a UWB-based RTLS.
- It does not require a reference system of predefined coordinates for placement coordination among anchor nodes.
- The measurement process does not require clock synchronization of the UWB nodes.

## 2. Node Calibration System

In this section, we provide the comprehensive mathematical modeling, design, and implementation of the proposed system. Based on the principle of multiple simultaneous ranging (MSR) [34] and the property of time difference of reception of two packets transmitted from different sources, we formulate the proposed system to simultaneously measure the position and antenna delay values of anchor nodes in a UWB-based RTLS.

### 2.1. Principle

Suppose that a wireless network comprises UWB nodes, where a mobile node M and an anchor node A transmit the first and second packets of a sensing session, called sensing packets. Let $P_{i}$ denote the time difference of reception of the two sensing packets at node *i*. Then, for any receptor nodes *i* and *j*, it can be shown that [34]

(1) $$P_{i}-P_{j}=\left(T_{P(A,i)}-T_{P(A,j)}\right)-\left(T_{P(M,i)}-T_{P(M,j)}\right),$$

where $T_{P(i,j)}$ is the TOF from node *i* to node *j*. Now, suppose that $P_{i}$ is to be computed as if it was measured by a preferred clock, and the clock of M is selected as the preferred clock. To facilitate this computation, let M transmit a third packet of the sensing session. Then, the ratio of the clock speed of M over the clock speed of *i* is given by

(2) $$r_{i}^{M}=\left\{\begin{array}{cc} 1, & \mathrm{if}\;i=M\\ \frac{t_{\mathrm{Tx}(3)}^{M}-t_{\mathrm{Tx}(1)}^{M}}{t_{\mathrm{Rx}(3)}^{i}-t_{\mathrm{Rx}(1)}^{i}}, & \mathrm{otherwise}, \end{array}\right.$$

where $t_{\mathrm{Tx}(n)}^{i}$ and $t_{\mathrm{Rx}(n)}^{i}$ denote, respectively, the transmission and reception timestamp values read by node *i* for the $n^{\mathrm{th}}$ packet. In addition, for nodes M, A, and any receptor anchor node *X*, the corresponding time difference of reception can be computed by

(3) $$\begin{array}{cc} P_{M} & =t_{\mathrm{Rx}(2)}^{M}-\left(t_{\mathrm{Tx}(1)}^{M}+d_{M}^{M}\right)\\ \; & =P_{M}^{M,U}-d_{M}^{M}, \end{array}$$

(4) $$\begin{array}{cc} P_{A} & =\left\{\left(t_{\mathrm{Tx}(2)}^{A}+d_{A}^{A}\right)-t_{\mathrm{Rx}(1)}^{A}\right\}r_{A}^{M}\\ \; & =\left\{t_{\mathrm{Tx}(2)}^{A}-t_{\mathrm{Rx}(1)}^{A}\right\}r_{A}^{M}+d_{A}^{A}r_{A}^{M}\\ \; & =P_{A}^{M,U}+d_{A}^{M}, \end{array}$$

and

(5) $$\begin{array}{cc} P_{X} & =\left\{t_{\mathrm{Rx}(2)}^{X}-t_{\mathrm{Rx}(1)}^{X}\right\}r_{X}^{M}\\ \; & =P_{X}^{M,U}, \end{array}$$

where $d_{i}^{j}$ is the aggregate transmitting and receiving antenna delays of node *i* measured with the clock of node *j*, and $P_{i}^{j,U}$ is the antenna-delay-uncalibrated time difference of reception of node *i* measured with the clock of node *j*.

Now, given $P_{M}$ and $P_{A}$, the TOF from node M to node A can be obtained from (1) as

(6) $$\begin{array}{cc} \; & T_{P(M,A)}=\frac{\left(P_{M}-P_{A}\right)}{2}. \end{array}$$

In addition, for any receptor anchor node *X*, an independent equation can be constructed based on (1) such that

(7) $$P_{M}-P_{X}=\left(T_{P(A,M)}-T_{P(A,X)}\right)-\left(T_{P(M,M)}-T_{P(M,X)}\right).$$

Substituting (6) into (7) and rearranging the terms, we obtain

(8) $$\begin{array}{c} T_{P(M,X)}-T_{P(A,X)}=\left(P_{M}-P_{X}\right)-\frac{\left(P_{M}-P_{A}\right)}{2}. \end{array}$$

Now, if X=A, substituting the corresponding time difference of reception values from (3) and (4) into (8), we get

(9) $$\begin{array}{cc} \; & T_{P(M,A)}+\frac{d_{M}^{M}}{2}+\frac{d_{A}^{M}}{2}=\frac{\left(P_{M}^{M,U}-P_{A}^{M,U}\right)}{2}. \end{array}$$

Similarly, if X≠A, substituting the corresponding time difference of reception values from (3), (4), and (5) into (8), we get

(10) $$\begin{array}{cc} \; & T_{P(M,X)}-T_{P(A,X)}+\frac{d_{M}^{M}}{2}-\frac{d_{A}^{M}}{2}=\frac{\left(P_{M}^{M,U}+P_{A}^{M,U}\right)}{2}-P_{X}^{M,U}. \end{array}$$

Multiplying both sides of (9) and (10) by the speed of light, *c*, the corresponding range between a pair of nodes *i* and *j*, denoted as R(i,j), and the antenna-delay-induced length of node *i*, denoted as $L_{i}$, is given by

(11) $$\begin{array}{cc} \; & R\left(M,A\right)+\frac{L_{M}}{2}+\frac{L_{A}}{2}=\beta_{A,A,M} \end{array}$$

and

(12) $$\begin{array}{cc} \; & R\left(M,X\right)-R\left(A,X\right)+\frac{L_{M}}{2}-\frac{L_{A}}{2}=\beta_{A,X,M} \end{array}$$

respectively, where

(13) $$\beta_{A,X,M}=\left\{\begin{array}{cc} c\times \left(\frac{\left(P_{M}^{M,U}-P_{A}^{M,U}\right)}{2}\right), & \mathrm{if}\;X=A\\ c\times \left(\frac{\left(P_{M}^{M,U}+P_{A}^{M,U}\right)}{2}-P_{X}^{M,U}\right), & \mathrm{if}\;X\neq A, \end{array}\right.$$

is the value computed from the antenna-delay-uncalibrated time difference of receptions of the respective nodes, hereinafter referred to as sensing information.

### 2.2. Linearization via Taylor Series

Consider a set of anchor nodes $A=\{A_{1},A_{2},\ldots ,A_{I}\}$ located at *I* fixed unknown locations and *K* known positions of a mobile node M with a fixed z−coordinate. Let $p_{i}=\left[x_{i},y_{i},z_{i}\right],\forall i\in \{1,\ldots ,I$} and $q_{k}=\left[x_{k},y_{k},z_{k}\right],\forall k\in \left\{1,\ldots ,K\right\}$ be the three-dimensional vectors of xyz-coordinates of the positions of the anchor and mobile nodes, respectively. Then, (11) and (12) can be written, respectively, as

(14) $$R\left(q_{k},p_{i}\right)+\frac{L_{M}}{2}+\frac{L_{A_{i}}}{2}=\beta_{i,i,k}$$

and

(15) $$R\left(q_{k},p_{j}\right)-R\left(p_{i},p_{j}\right)+\frac{L_{M}}{2}-\frac{L_{A_{i}}}{2}=\beta_{i,j,k},$$

where i≠j,1≤i≤I,1≤j≤I, and 1≤k≤K.

Equations (14) and (15) are non-linear with $I^{2}K$ independent equations and 4I+1 unknown variables, i.e., the xyz-coordinates of unknown positions of the anchor nodes and the antenna-delay-induced length of the anchor nodes and the mobile node. They can be expressed as a general form

(16) f(x)=B,

where x is a vector of the unknown variables and *B* is the sensing information. Now, we use Taylor series expansion of (16) to linearize the function and obtain the Taylor series least-squares (TSLS) solutions by iteration. The Taylor series expansion of (16) is given by

(17) $$\begin{array}{c} f\left(x\right)≃f\left(x_{n}\right)+{\left.\frac{\partial f}{\partial x}\right|}_{x=x_{n}}\Delta x_{n}=B, \end{array}$$

where $x_{n}$ is a vector of approximate values of the variables at the $n^{\mathrm{th}}$ iteration, and $\Delta x_{n}$ is a vector of corrections to the approximate values of the variables at the $n^{\mathrm{th}}$ iteration. TSLS finds the least-squares solutions of (16) by iterating

(18) $$x_{n+1}=x_{n}+\Delta x_{n},$$

where $\Delta x_{n}$ is the linear least-squares solution of (17).

### 2.3. System Design and Implementation

The system for node calibration is designed to estimate the position and antenna delay values of UWB anchor nodes that are located at fixed unknown locations. The system is set to operate as follows. The setup includes a set of UWB anchor nodes $A=\{A_{1},A_{2},\ldots ,A_{I}\}$ that are located at *I* fixed unknown locations and a UWB mobile node M that is moved around and placed at *K* known positions that ensure LOS with the anchor nodes. The transmission and reception of packets to and from the anchor and mobile nodes are over the air, i.e., these nodes are fully wireless. The system further includes two complementary entities: a UWB-based master node and a computer (PC). The master node operates to request initiation of a sensing session, collect components of the sensing information from the other UWB nodes, i.e., the antenna-delay-uncalibrated time difference of receptions of respective nodes, and log the data to the PC via a serial UART. At each of the *K* known positions, the mobile node performs TWR sessions based on MSR [34], where the mobile node is the selected node of the preferred clock and the transmitter of the first and third packets during a sensing session, with all the anchor nodes. Hence, each sensing session yields one sensing information based on (11) and (I−1) number of sensing information based on (12). The components of the sensing information are hereinafter referred to as session-data. After completion of a predefined number of sensing sessions, the collected sets of session-data are processed at the PC, where the position and antenna delay values of the anchor nodes are estimated as the TSLS solutions.

The node calibration measurement process follows the following sequence.

Step 1: Place each of the anchor nodes (powered) at their respective locations, $p_{i}=\left[x_{i},y_{i},z_{i}\right],\forall i\in \left\{1,\ldots ,I\right\}$.
Step 2: Identify the known positions of mobile nodes, $q_{k}=\left[x_{k},y_{k},z_{k}\right],\forall k\in \left\{1,\ldots ,K\right\}$, such that they have a clear LOS with the anchor nodes and $K\geq \frac{4I+1}{I^{2}}$.
Step 3: Session-data collection at mobile node positions, k=(1,…,K).
  Step 3.1: Place the mobile node M at the *k*-th position.
  Step 3.2: The PC requests the start of sensing sessions from the master node.
  Step 3.3: The master node initiates a sensing session based on MSR, where it assigns M to transmit the first and third sensing packets, $A_{i}$ to transmit the second sensing packet, and $A\setminus A_{i}$ to listen to the three sensing packets. The master node then collects the session-data from each respective node after a successful sensing session. The process of Step 3.3 is repeated for i=(1,…,I) sensing sessions, and the sets of session-data are stored in the PC as a log file.
  Step 3.4: The process of Step 3.2 to Step 3.3 is repeated until 1000 sets of log files have been stored in the PC.
Step 4: The processing of the session-data is performed at the PC, where the xyz-coordinates of unknown positions of the anchor nodes and the antenna-delay-induced length of the anchor nodes and the mobile node are estimated as the TSLS solutions of (17).

The implementation of each entity in the system was based on the cooperation mentioned above. The UWB nodes were Decawave’s DW1000 UWB transceiver [35], which complies with the IEEE 802.15.4-2011 standard [19]. The operation of the UWB nodes was implemented in the C programming language using Segger Embedded Studio [36] as an integrated development environment, and the PC was implemented using Python programming.

## 3. Numerical Results

### 3.1. Experimental Evaluation

In this section, we present an experimental evaluation of the proposed system. Considering a TWR-based RTLS application in an indoor environment, at least four nearby anchors, those not on the same plane, are considered to estimate a three-dimensional position of a mobile node. A crucial consideration for anchor placement is to avoid NLOS conditions between mobile and nearby anchors, since they would induce positive biases on the estimated TOFs that may vary dynamically and significantly as the mobile moves around. Several NLOS mitigation techniques are present in the literature [37,38,39]. However, a TOF estimated from NLOS conditions cannot have LOS-equivalent results. Hence, to ensure that a TOF estimated from NLOS conditions is rarely used for position estimation, we assume a rule of anchor placement where a mobile node in the coverage area should typically find LOSs to at least four nearby anchors.

**Figure 1:**
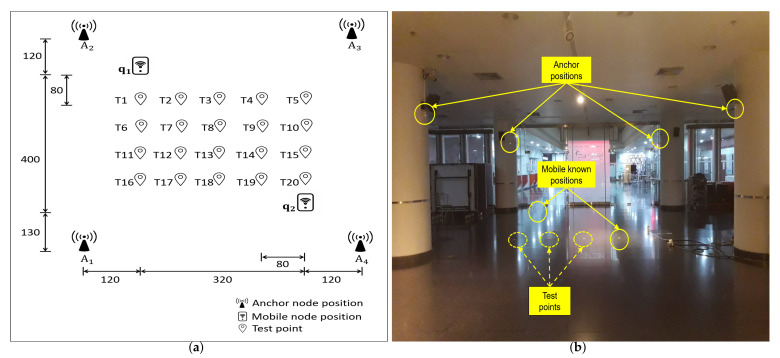
(**a**) Experimental layout (unit: cm). The positions of the anchor nodes, the known positions of the mobile node, and the test points for the mobile node. (**b**) Measurement and test environment.

In this regard, the experimental setup included a master node, four anchor nodes, a mobile node, and a PC. Each of these nodes was based on Decawave’s DW1000 UWB transceiver [35]. The four anchor nodes to be calibrated were mounted on pillars in an indoor hallway, as shown in Figure 1. Let the address identification of the four anchor nodes be $A_{1},A_{2},A_{3},$ and $A_{4}$. In addition, two points were identified at ground level to be the known positions of the mobile node M such that they had a clear LOS with the four anchor nodes. The coordinates (unit: cm) of the known positions of the mobile node were identified as $q_{1}=\left(320,0,0\right)$ and $q_{2}=\left(0,400,0\right)$, as shown in Figure 1. Then, based on the above experimental setup and following the measurement process detailed in Section 2.3, we performed the calibration of the anchor nodes. The measurement results show that the positions (unit: cm) of the four anchor nodes were $A_{1}=\left(444.99,541.36,224.57\right)$, $A_{2}=\left(448.98,-78.8,216.44\right),$$A_{3}=\left(-132.42,-118.46,195.31\right),$ and $A_{4}=\left(-143.76,546.57,206.83\right)$, and the antenna-delay-induced lengths (unit: cm) of the four anchor nodes and the mobile node were $L_{A_{1}}=-12.92,L_{A_{2}}=14.77,L_{A_{3}}=-21.59,L_{A_{4}}=-48.66$, and $L_{M}=12.32$, respectively.

Then, the accuracy of the proposed system was evaluated by using it to localize a mobile node at a number of test points in LOS coverage with the four anchor nodes. Here, we considered three calibration cases of anchor nodes: a pre-calibrated case where both the position and antenna delay values of the anchor nodes were roughly guessed; a post-calibrated (with antenna delay) case where both the position and antenna delay values of the anchor nodes were obtained from the measurement process; and a post-calibrated (without antenna delay) case where only the position values of the anchor nodes were obtained from the measurement process while the antenna delay values were roughly guessed. A set of 20 test points were identified in the indoor hallway, denoted as T1,T2,…, and T20, as shown in Figure 1a, where the mobile node was to be localized. The mobile node was placed at each test point, where 1000-sample ranges were obtained for each possible pair of the mobile and anchor nodes for two existing state-of-the-art TWR methods: alternative double-sided TWR (AltDS-TWR) [23] and MSR [34], where the mobile node was the selected node of the preferred clock and the transmitter of the first and third packets during a sensing session. Finally, the positions of the mobile node were estimated at each test point as the TSLS solutions, and root-mean-square errors (RMSEs) were computed to analyze the accuracy of the three calibration cases.

#### 3.1.1. Numerical Results for AltDS-TWR

**Figure 2:**
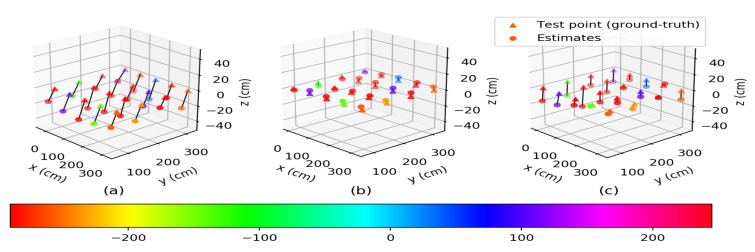
Means of the estimated positions of the mobile node at each test point from AltDS-TWR for the three calibration cases of anchor nodes: (**a**) pre-calibrated case, (**b**) post-calibrated (with antenna delay) case, and (**c**) post-calibrated (without antenna delay) case.

Figure 2 shows the mean of the estimated positions of the mobile node at each test point from AltDS-TWR for the three calibration cases of anchor nodes. As seen in Figure 2b,c, the means of the estimated positions for the two post-calibrated cases were closer to the ground-truths (indicating the test points) compared to the pre-calibrated case in Figure 2a. Moreover, from the plots in Figure 2b, we can see that the estimated positions were approximately superimposed over the ground-truths. This suggests the effectiveness of the post-calibrated (with antenna delay) case over the pre-calibrated and post-calibrated (without antenna delay) cases. The localization errors for the three calibration cases of anchor nodes are summarized in Table 1.

The RMSEs (unit: cm) of the estimated xyz-coordinates and positions of the mobile node at each test point from AltDS-TWR for the three calibration cases of anchor nodes are given in Table 1. For the pre-calibrated case, the average RMSEs of the estimated xyz-coordinates for all of the test points were 16.56,10.6, and 20.75, respectively. For the post-calibrated (with antenna delay) case, the average RMSEs of the estimated xyz-coordinates for all of the test points were 2.9,2.58, and 3.92, respectively. For the post-calibrated (without antenna delay) case, the average RMSEs of the estimated xyz-coordinates for all of the test points were 3.26,3.53, and 12.53, respectively. From these analyses of the estimated results of the xyz-coordinates in Table 1, we may conclude that the post-calibrated (with antenna delay) case showed a desirable performance improvement in estimating all three coordinates of the mobile node at the test points. For the estimated positions in Table 1, the maximum RMSEs were 36.76,8.2, and 25.59, while the minimum RMSEs were 20.12,2.64, and 8.81 for the pre-calibrated, post-calibrated (with antenna delay), and post-calibrated (without antenna delay) cases, respectively. For all of the test points, the RMSEs for the two post-calibrated cases were significantly reduced compared to those of the pre-calibrated case. The averages of the RMSEs for all of the test points were 29.78,5.94, and 13.78 for the pre-calibrated, post-calibrated (with antenna delay), and post-calibrated (without antenna delay) cases, respectively. Here, the post-calibrated (with antenna delay) case had RMSE reductions of approximately 80.05% and 56.89% compared to the pre-calibrated and post-calibrated (without antenna delay) cases, respectively. This further signifies that the post-calibrated (with antenna delay) case of anchor nodes was effective in localizing the mobile node at each of the test points.

#### 3.1.2. Numerical Results for MSR

**Figure 3:**
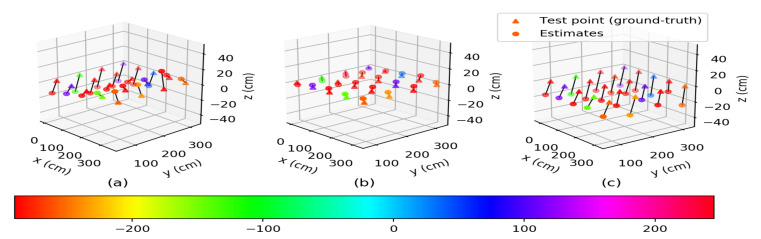
Mean of the estimated positions of the mobile node at each test point from MSR for the three calibration cases of anchor nodes: (**a**) pre-calibrated case, (**b**) post-calibrated (with antenna delay) case, and (**c**) post-calibrated (without antenna delay) case.

Figure 3 shows the means of the estimated positions of the mobile node at each test point from MSR for the three calibration cases of anchor nodes. Similarly to the plots in Figure 2, the means of the estimated positions for the two post-calibrated cases, as seen in Figure 3b,c, were closer to the ground-truths compared to those of the pre-calibrated case in Figure 3a. In addition, as shown in the plots in Figure 3b, the estimated positions were approximately superimposed over the ground-truths. This suggests the effectiveness of the post-calibrated (with antenna delay) case over the pre-calibrated and post-calibrated (without antenna delay) cases. The localization errors for the three calibration cases of anchor nodes are summarized in Table 2.

The RMSEs (unit: cm) of the estimated xyz-coordinates and positions of the mobile node at each test point from MSR for the three calibration cases of anchor nodes are given in Table 2. For the pre-calibrated case, the average RMSEs of the estimated xyz-coordinates for all of the test points were 16.85,26.86, and 14.44, respectively. For the post-calibrated (with antenna delay) case, the average RMSEs of the estimated xyz-coordinates for all of the test points were 3.49,3.18, and 8.31, respectively. For the post-calibrated (without antenna delay) case, the average RMSEs of the estimated xyz-coordinates for all of the test points were 9.43,8.85, and 20.43, respectively. From these analyses of the estimated results of the xyz-coordinates in Table 2, we may conclude that the post-calibrated (with antenna delay) case showed a desirable performance improvement in estimating all three coordinates of the mobile node at the test points. For the estimated positions in Table 2, the maximum RMSEs were 53.48,16.66, and 29.91, while the minimum RMSEs were 26.38,5.91, and 16.59 for the pre-calibrated, post-calibrated (with antenna delay), and post-calibrated (without antenna delay) cases, respectively. For all of the test points, the RMSEs for the two post-calibrated cases were significantly reduced compared to that of the pre-calibrated case. The averages of the RMSEs for all the test points were 35.53,9.74, and 24.64 for the pre-calibrated, post-calibrated (with antenna delay), and post-calibrated (without antenna delay) cases, respectively. Here, the post-calibrated (with antenna delay) case had RMSE reductions of approximately 72.59% and 60.47% with respect to the pre-calibrated and post-calibrated (without antenna delay) cases, respectively. This further signifies that the post-calibrated (with antenna delay) case of anchor nodes was effective in localizing the mobile node at each of the test points for MSR as well.

From these analyses of the test results for the two TWR methods, we can conclude that the proposed system can effectively estimate the position and antenna delay values of anchor nodes, where the post-calibrated (with antenna delay) case of anchor nodes can provide more accurate localization of a mobile node. For the post-calibrated (with antenna delay) anchor nodes, the RMSEs of the estimated xyz-coordinates and positions of the mobile node for both TWR methods were within the same order of magnitude and with centimeter-level accuracy. Hence, the proposed system should be applicable for the calibration of anchor nodes in the current accuracy-demanding UWB-based RTLS applications. Finally, in Table 3, we summarize by comparing our results with those of other state-of-the-art auto-calibration methods, which mainly differ in providing simultaneous measurement of the position and antenna delay values of the anchor nodes during the calibration process.

## 4. Conclusions

This paper presented a novel node calibration system that simultaneously measures the position and antenna delay values of the anchor nodes in UWB-based RTLSs. Based on an experiment comparing the accuracy when localizing a mobile node at a number of test points satisfying LOS conditions, the post-calibrated (with antenna delay) case of anchor nodes whose position and antenna delay values were measured by the proposed system provided more accurate location estimations. The results show the significance of the proposed system in maintaining a highly effective UWB-based RTLS.

## Figures and Tables

**Table 1:** Comparison of the RMSEs (unit: cm) of the estimated xyz-coordinates and positions of the mobile node at each test point from AltDS-TWR for the pre-calibrated, post-calibrated (with antenna delay), and post-calibrated (without antenna delay) cases of anchor nodes.

Test Point	RMSE (cm)											
	Pre-Calibrated				Post-Calibrated				Post-Calibrated			
					(with Antenna Delay)				(without Antenna Delay)			
	Coordinates			Position	Coordinates			Position	Coordinates			Position
	x-	y-	z-		x-	y-	z-		x-	y-	z-	
T1	6.19	15.27	18.19	24.54	1.68	6.48	3.03	7.35	1.51	2.91	9.08	9.65
T2	14.35	13.34	22.47	29.81	4.47	4.18	2.19	6.5	3.1	2.66	10.01	10.81
T3	19.34	9.75	25.47	33.43	1.78	1.05	7.07	7.37	2.17	4.31	7.37	8.81
T4	26.08	7.41	23.36	35.79	1.45	1.16	4.11	4.51	1.18	7.53	13.75	15.72
T5	28.58	6.35	18.55	34.66	3.6	1.17	2.3	4.44	2.12	5.22	15.94	16.91
T6	8.01	15.37	19.86	26.36	4.71	1.52	5.26	7.23	5.91	2.08	6.33	8.9
T7	7.49	10.98	21.36	25.16	2.52	1.26	5.68	6.34	1.73	2.16	8.72	9.15
T8	15.19	8	24.82	30.18	1.24	3.38	6.06	7.05	2.61	2.03	11.89	12.34
T9	21.72	9.08	28.24	36.76	1.46	1.36	3.07	3.66	4.46	8.03	23.89	25.59
T10	26.84	7.76	20.36	34.57	2.59	0.98	4.44	5.23	1.62	3.08	17.87	18.2
T11	2.03	16.4	11.49	20.12	1.76	1.26	1.5	2.64	1.75	1.14	13.04	13.21
T12	7.43	13.88	17.63	23.64	4.39	3.23	5.94	8.06	1.18	1.18	12.34	12.45
T13	17.27	9.51	17.98	26.68	4.49	2.24	3.48	6.11	3.82	3.11	12.8	13.71
T14	19.85	8.66	20.75	29.99	1.18	2.31	5.5	6.08	2.23	2.37	14.34	14.71
T15	26.47	7.84	18.95	33.49	1.28	1.55	3.43	3.98	4.7	2.32	13.24	14.24
T16	6.55	16.7	21.68	28.14	4.72	1.34	4.59	6.72	2.44	1.67	11.06	11.45
T17	8.6	10.17	16.75	21.39	3.51	6.73	3.03	8.17	2.1	5.66	14.29	15.51
T18	19.06	8.99	26.67	33.99	5.63	4.73	3.63	8.2	7.73	5.6	10.01	13.83
T19	23.31	8.03	23.56	34.1	4.04	3.9	2.33	6.07	8.96	5.98	8.26	13.57
T20	26.93	8.52	16.86	32.89	1.63	1.84	1.82	3.06	3.8	1.62	16.29	16.81

**Table 2:** Comparison of RMSEs (unit: cm) of the estimated xyz-coordinates and positions of the mobile node at each test point from MSR for the pre-calibrated, post-calibrated (with antenna delay), and post-calibrated (without antenna delay) cases of anchor nodes.

Test Point	RMSE (cm)											
	Pre-Calibrated				Post-Calibrated				Post-Calibrated			
					(with Antenna Delay)				(without Antenna Delay)			
	Coordinates			Position	Coordinates			Position	Coordinates			Position
	x-	y-	z-		x-	y-	z-		x-	y-	z-	
T1	21.26	26.2	18.79	38.62	3.23	3.85	10.3	11.47	6.99	15.32	17.38	24.2
T2	14.97	26.36	10.18	31.98	3.49	4.19	9.99	11.38	9.96	15.44	13.94	23.07
T3	12.19	24.29	5.04	27.64	2.59	2.75	8.16	8.99	10.6	13.06	16.19	23.35
T4	9.33	23.31	8.07	26.38	2.3	2.55	5.26	6.28	11.69	12.22	15.31	22.82
T5	9.93	22.96	13.7	28.52	4.64	2.62	5.16	7.42	9.34	12.71	17.57	23.61
T6	21.63	29.13	19.56	41.22	4.91	2.83	5.91	8.19	10.35	11.11	23.39	27.89
T7	23.07	25.13	11.47	35.99	3.58	2.83	9.22	10.29	6.15	7.77	18.47	20.96
T8	17.77	23.13	5.27	29.65	2.99	3.83	6.76	8.33	8.57	7.05	19.05	22.04
T9	14.23	24.92	14.84	32.3	3.18	2.94	5.82	7.25	9.8	10.13	23.27	27.21
T10	11.33	24.25	19.8	33.29	3.99	2.59	6.51	8.07	10.52	11.46	23.69	28.34
T11	30.55	32.62	29.37	53.48	3.6	2.23	14.79	15.38	4.41	6.98	17.73	19.55
T12	24.88	30.97	12.06	41.51	3.15	2.29	13.85	14.38	5.79	7.3	18.14	20.39
T13	15.52	27.11	5.14	31.66	3.88	3.08	13.05	13.96	11.77	6.44	17.2	21.82
T14	14.82	26.05	14.42	33.26	3.14	3.09	7.46	8.66	10.21	8.02	22.5	25.98
T15	11.28	24.86	21.99	35.05	3.16	2.64	7.21	8.31	11.52	9.47	25.93	29.91
T16	25.7	36.78	12.42	46.55	5.01	2.14	7.31	9.12	7.77	4.21	25.26	26.77
T17	23.7	29.51	10.5	39.28	2.92	6.04	15.25	16.66	5.89	3.04	15.21	16.59
T18	14.81	28.27	14.66	35.12	4.37	4.63	4.97	8.08	11.31	3.49	26.33	28.87
T19	11.12	26.02	19.62	34.43	3.2	3.68	4.67	6.75	12.73	4.62	26.38	29.65
T20	8.93	25.31	21.91	34.65	2.5	2.7	4.62	5.91	13.32	7.08	25.75	29.84

**Table 3:** Comparison of auto-calibration methods for UWB-based anchor nodes.

Method	Result	Clock	Reference	Calibration	RMSE (cm)
		Synchronization	System		
Hamer et al. [28]	Experiment	Required	Required	Position	9.7
Vashistha et al. [29]	Experiment	Required	Required	Position	30
Almansa et al. [30]	Simulation	Not required	Not required	Position	135
Proposed	Experiment	Not required	Not required	Position and	5.94
				antenna delay	

## Data Availability

Not applicable.
